# Generation of 3 patient induced Pluripotent stem cell lines containing *SORD* mutations linked to a recessive neuropathy

**DOI:** 10.1016/j.scr.2024.103449

**Published:** 2024-05-22

**Authors:** Christopher Yanick, Renata Maciel, Elizabeth Jacobs, Jacquelyn Schatzman, Michael Shy, Stephan Zuchner, Mario Saporta

**Affiliations:** a Department of Neurology, University of Miami Miller School of Medicine, Miami, FL, United States; b Hussman Institute for Human Genomics, University of Miami Miller School of Medicine, Miami, FL, United States; c University of Iowa Carver College of Medicine, Iowa City, Iowa, United States

## Abstract

The *SORD* neuropathy has been identified as the most common autosomal recessive inherited neuropathy, occurring in thousands of patients worldwide. Fibroblast lines from 3 different patients containing the c.753delG; p.Ala253GlnfsTer27 *SORD* mutations were reprogrammed into induced Pluripotent Stem Cell (iPSC) lines. These iPSC lines demonstrate an apparent normal karyotype and have positive expression of pluripotency markers. These iPSC lines also stain positively for Ectoderm, Endoderm and Mesoderm markers following Embryoid body differentiation. These lines pose to serve as a valuable disease modeling resource for studying the *SORD* neuropathy, including studying disease phenotype and treatment efficacy.

## 1. Resource Utility

Estimates of patients worldwide with the *SORD* neuropathy are currently in the thousands. While an ongoing clinical trial is investigating efficacy of Aldose Reductase inhibition (NCT05397665), more needs to be done to study phenotype. These iPSCs give an outlet to study treatments, and disease phenotype in a human cell model.Table 1..

## 2. Resource Details

The *SORD* neuropathy is caused by biallelic mutations to the gene *SORD*, encoding sorbitol dehydrogenase, leading to the arrest of the polyol pathway and the development of a peripheral neuropathy (Cortese, 2020). This recessive neuropathy is also the focus on an ongoing clinical trial looking at the efficacy of Aldose Reductase Inhibitor (ARI) treatment in patients (NCT05397665). Currently there are several thousand patients identified with these mutations and with the ongoing trial, the need to study and understand cellular phenotypes is ever growing.

**Fig. 1.**
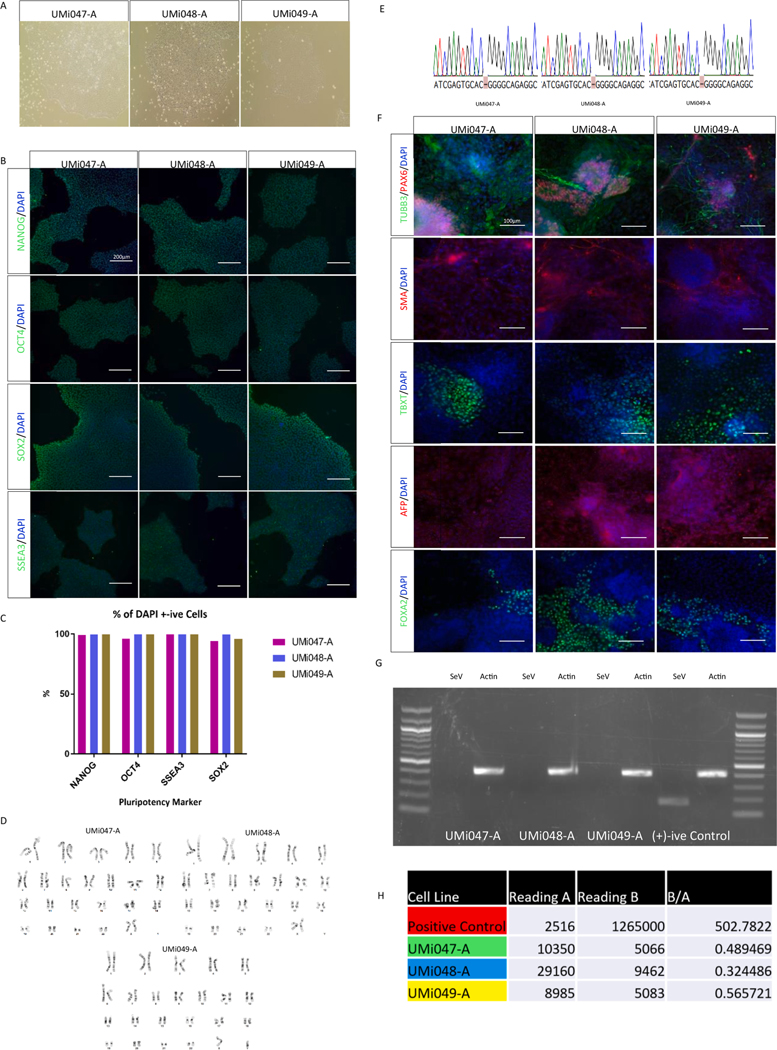


The 3 patient fibroblast lines containing the biallelic *SORD* mutations were plated into duplicate 6-well plates and grown to around 60 % confluency. The duplicate plates were counted prior to the reprogramming protocol to calculate for virus titers. The fibroblasts were transduced using the Sendai Virus 2.0 Reprogramming kit, containing vectors encoding *KLF4*, *C-Myc*, *OCT3/4* and *SOX2*. After 7 days, cells were replated onto 10 cm^2^ plates and allowed to grow with daily media changes. Following approximately 3–4 weeks of culturing and allowing colonies to form, several potential colonies were then chosen for each line and expanded (Table 2). A single colony for each line was then selected and characterized. Brightfield imaging of iPSC lines shows apparent normal iPSC morphology (Fig. 1, Panel A). Pluripotency staining for NANOG, SSEA3, OCT4 and SOX2 was performed, with each line showing successful expression of the markers (Fig. 1, Panel B). Each of these markers shows a high rate of staining efficiency (Fig. 1, Panel C), indicating successful reprogramming.

Karyotyping performed by Cell Line Genetics shows all iPSC lines have an apparent normal karyotype (Fig. 1, Panel D, Supplementary File 1). STR analysis was performed on the lines and confirmed their origins from the patient fibroblast lines (Supplementary File 2). All 3 lines were sequenced for the *SORD* gene and the mutations were confirmed present in each patient line (Fig. 1, Panel E). iPSC lines underwent embryoid body differentiation to demonstrate differentiation potential. Each of the lines show successful staining for Ectoderm (TUBB3, PAX6), Mesoderm (TBXT, SMA), and Endoderm (FOXA2, AFP) markers, demonstrating their potential to be differentiated. (Fig. 1, Panel F).

RT-PCR of iPSC lines for SeV based vectors shows successful removal of SeV vectors from the 3 iPSC lines (Fig. 1, Panel G), using an early passage (passage 3) clone as a positive PCR control. Mycoplasma testing of the lines confirmed that no mycoplasma species are present (Fig. 1, Panel H).

In ongoing studies in our lab, these cells have been successfully differentiated into Lower Motor Neurons reproducibly, following an in-house differentiation protocol (Saporta 2015), further demonstrating their differentiation potential and usefulness as a resource in studying the *SORD* neuropathy. The differentiated cells also replicate an important disease phenotype, increased intracellular sorbitol content, that is seen in patients and animal models (Zhu, 2023).

## 3. Materials and Methods

### 3.1. Reprogramming from human fibroblasts

Donor fibroblasts containing the same biallelic *SORD* mutations were grown to 60 % confluency in 6-well plate format using DMEM containing 10 % FBS. These fibroblasts were transduced using the Cytotune 2.0 Sendai Virus kit, with wells plated in parallel used to calculate appropriate viral titer. Cells were then replated into 10 cm^2^ dishes and cultured until colony formation was noticed. Colonies were selected based off morphology and plated into dishes coated with Matrigel. Media was transitioned to mTeSR + . Cells were passaged using 0.5 mM EDTA at a 1:5 ratio. Cells were grown at 37°C and 5 % CO_2_.

### 3.2. Immunocytochemistry

iPSCs (passage 13) were fixated using 4 % paraformaldehyde at room temperature for 15 min, permeabilized using 0.2 % PBS-T (Triton X-100) and blocked with 5 % BSA in PBS. Primary antibodies were diluted in 5 % BSA and incubated at 4C overnight. Secondary antibodies were diluted in 5 % BSA and incubated for 1 h at room temperature. DAPI was applied for 15 min and cells were then covered with PBS prior to imaging. Cells stained for AFP and SMA were fixated using cold methanol for 15 min prior to permeabilization.

### 3.3. Karyotype and STR analysis

Cells at passage 14 were shipped to Cell Line Genetics for karyotype analysis. iPSC cells were pelleted from culture, along with the parent fibroblast lines, with DNA extraction and STR analysis being performed by the University of California Berkeley DNA Sequencing Facility.

### 3.4. Analysis of mutations

DNA was isolated using the Qiagen DNeasy kit and an in-house PCR protocol for the *SORD* gene was performed to amplify fragments. PCR products were sent to Eurofins Scientific for Sanger Sequencing, with data being analyzed in our lab. We were able to confirm that all iPSC lines contained the biallelic *SORD* mutations.

### 3.5. Embryoid body differentiation

iPSCs (passage 15) were grown to 60–70 % confluency in 10 cm^2^ plates before being split into untreated 10 cm^2^ plates to allow a single cell suspension. Cells were then grown in Embryoid Body media (KO DMEM, 20 % KOSR, 1 % Glutamax, 1 % NEAA) with 10 μM Rock Inhibitor Y27632 and 100 μM Beta-mercaptoethanol, with media being changed every 48 h. This was done until Day 8, where the cell culture was pelleted and split into 96well plates treated with Matrigel for attachment, with the plates being fixed with 4 % paraformaldehyde after 48 h.

### 3.6. Mycoplasma testing

iPSC lines (passage 15) were plated in 6-well format and media was collected following 72 h of culture. Media was tested using the MycoAlert PLUS Mycoplasma Detection Kit (LONZA) including the MycoAlert Assay Control Set as a positive control.

### 3.7. RT-PCR for plasmids

RNA was extracted from iPSCs (passage 11) using the Qiashredder for lysing cells and the Qiagen RNEasy Plus Mini Kit. cDNA was generated using the QuantiTect Reverse Transcription. PCR was performed using the Platinum II Taq Hot-Start DNA Polymerase kit. PCR products were run on 1 % Agarose gels. SeV primers were used to test for presence of all 3 vectors, β-actin primers were used as a housekeeping/positive PCR control.

## Supplementary Material

1

## Figures and Tables

**Table 1:** Characterization Details.

Classification	Test	Result	Data
**Morphology**	Photography Bright field	*Normal*	Figure 1 *Panel A*
**Phenotype**	Qualitative analysis	*ICC for OCT4, NANOG, SSEA3 and SOX2*	Figure 1 *Panel B*
	Quantitative analysis	*OCT4: 96.17, 99.97, 99.97 NANOG: 99.44, 99.67, 99.99 SSEA3: 99.98, 99.97, 99.95 SOX2: 94.12, 99.63, 96.05*	Figure 1 *Panel C*
**Genotype**	Karyotype (G-banding) and resolution	*46XX, 46XX, 46XY*	Figure1 *Panel D*
**Identity**	Microsatellite PCR (mPCR) OR	*N/A*	*N/A*
	STR analysis	*iPSCs match fibroblast origin lines at 8 loci* + *amylogenin*	*With authors*
**Mutation analysis (IF APPLICABLE)**	Sequencing	*Homozygous*	Figure 1 *Panel E*
	Southern Blot OR WGS	*N/A*	*N/A*
**Microbiology and virology**	Mycoplasma	*Mycoplasma testing by luminescence. Negative result*	Figure 1 *Panel H*
**Differentiation potential**	e.g. Embryoid body formation OR Teratoma formation OR Scorecard OR Directed differentiation	*Embryoid body differentiation. Lower motor neuron directed differentiation performed in subsequent studies*	Figure 1 *Panel F*
**List of recommended germ layer markers**	**Expression of these markers has to be demonstrated at mRNA (RT PCR) or protein (IF) levels, at least 2 markers need to be shown per germ layer**	Endoderm: FOXA2, AFP Ectoderm: PAX6, TUBB3 Mesoderm: A-SMA, TBXT	Figure 1 *Panel F*
**Donor screening (OPTIONAL)**	HIV 1 + 2 Hepatitis B, Hepatitis C	*N/A*	N/A
**Genotype additional info (OPTIONAL)**	Blood group genotyping	*N/A*	N/A
	HLA tissue typing	*N/A*	N/A

**Table 2:** Reagent Details.

	Antibodies used for immunocytochemistry/flow-cytometry			
	
	Antibody	Dilution	Company Cat #	RRID
Pluripotency	Mouse anti-SSEA3 (MC-631)	1:100	Thermo Fischer Scientific Cat# MA1–020-D488	AB_2536683
	Mouse anti-NANOG	1:100	EMD Millipore Cat# MABD24A4	AB_2847877
	Mouse anti-OCT4 (POUF5F1)	1:100	EMD Millipore Cat# MAB4419A4	AB_2847875
	Rabbit anti-SOX2	1:100	EMD Millipore Cat# AB5603	AB_2286686
Differentiation Potential − Ectoderm	Rabbit anti-β-Tubulin III	1:500	Sigma-Aldrich Cat#T2200	AB_262133
	Mouse anti-Pax6	1:200	Santa Cruz Biotechnology Cat# sc-81649	AB_1127044
Differentiation Potential − Mesoderm	Mouse anti-Smooth Muscle Actin (B4)	1:100	Santa Cruz Biotechnology Cat# sc-53142d	AB_2273670
	Rabbit anti-Brachyury (D2Z3J)	1:1000	Cell Signaling Technology Cat# 81,694	AB_2799983
Differentiation Potential − Endoderm	Mouse anti-AFP (C3)	1:100	Santa Cruz Biotechnology Cat# sc-8399	AB_626665
	Rabbit anti-FoxA2/HNF3β	1:400	Cell Signaling Technology Cat# 8186	AB_10891055
Secondary antibodies	Goat anti-Rabbit IgG (H + L) Highly Cross-Adsorbed Secondary Antibody, Alexa Fluor^™^ Plus 488	1:400	Thermo Fisher Scientific Cat# A32731	AB_2633280
	Goat anti-Mouse IgG (H + L) Highly Cross-Adsorbed Secondary Antibody, Alexa Fluor^™^ Plus 594	1:400	Thermo Fisher Scientific Cat# A32742	AB_2762825

**Table:** 4. Resource Table:

Unique stem cell lines identifier	*UMi047-AUMi048-AUMi049-A*
Alternative name(s) of stem cell lines	*sord 1 DM (UMi047-A)sord 1 GB (UMi048-A) IIsord1 RV (UMi049-A)*
Institution	*University of Miami Miller School of Medicine*
Contact information of distributor	*Christopher Yanick;* cxy256@miami.eduMario *Saporta;* mas638@med.miami.edu
Type of cell lines	*iPSC*
Origin	*human*
Additional origin info required for human ESC or iPSC	*UMi047-A: 37, Female, German/SwedishUMi048- A: 71, Female, English/Romanian (Ashkenazi Jewish)UMi049-A: 20, Male, Italian/Irish*
Cell Source	*Dermal fibroblasts*
Clonality	*Clonal*
Method of reprogramming	*Sendai virus transduction*
Genetic Modification	*Yes*
Type of Genetic Modification	*Hereditary*
Evidence of the reprogramming transgene loss (including genomic copy if applicable)	*RT-PCR,* Figure 1 *Panel G*
Associated disease	*SORD neuropathy; sorbitol dehydrogenase deficiency with peripheral neuropathy*
Gene/locus	*SORD; Sorbitol Dehydrogenase: chromosome 15c.753delG; p.Ala253GlnfsTer27*
Date archived/stock date	*N/A*
Cell line repository/bank	https://hpscreg.eu/cell-line/UMi047-Ahttps://hpscreg.eu/cell-line/UMi048-Ahttps://hpscreg.eu/cell-line/UMi049-A.
Ethical approval	*Ethical approval was provided through the Inherited Neuropathy Consortium at the University of Pennsylvania (IRB# 832955) as well as the University of Iowa (IRB# 201201787).*

## References

[R1] Cortese A, Zhu Y, Rebelo AP, Negri S, Courel S, Abreu L, Bacon CJ, Bai Y, Bis-Brewer DM, Bugiardini E, Buglo E, Danzi MC, Feely SME, Athanasiou-Fragkouli A, Haridy NA, Inherited Neuropathy Consortium, Isasi R, Khan A, Laurà M, Magri S, Pipis M, Pisciotta C, Powell E, Rossor AM, Saveri P, Tozza Sowden J.E., Vandrovcova SJ, Dallman J, Grignani E, Marchioni E, Scherer SS, Tang B, Lin Z, Al-Ajmi A, Schüle R, Synofzik M, Maisonobe T, Stojkovic T, Auer-Grumbach M, Abdelhamed MA, Hamed SA, Zhang R, Manganelli F, Santoro L, Taroni F, Pareyson D, Houlden H, Herrmann DN, Reilly MM, Shy ME, Zhai RG, Zuchner S, 2020 May. Biallelic mutations in SORD cause a common and potentially treatable hereditary neuropathy with implications for diabetes. Nat Genet. 52 (5), 473–481. 10.1038/s41588-020-0615-4. Epub 2020 May 4. Erratum. In: Nat Genet. 2020 Jun; 52(6):640. PMID: 32367058; PMCID: PMC8353599. 32367058 PMC8353599

[R2] Saporta MA, Dang V, Volfson D, Zou B, Xie XS, Adebola A, Liem RK, Shy M, Dimos JT. Axonal Charcot-Marie-Tooth disease patient-derived motor neurons demonstrate disease-specific phenotypes including abnormal electrophysiological properties. Exp Neurol. 2015 Jan;263:190–9. doi: 10.1016/j.expneurol.2014.10.005. Epub 2014 Oct 30. PMID: 25448007; PMCID: PMC4262589. 25448007 PMC4262589

[R3] Zhu Y, Lobato AG, Rebelo AP, Canic T, Ortiz-Vega N, Tao X, Syed S, Yanick C, Saporta M, Shy M, Perfetti R, Shendelman S, Züchner S, Zhai RG, 2023 May 22. Sorbitol reduction via govorestat ameliorates synaptic dysfunction and neurodegeneration in sorbitol dehydrogenase deficiency. JCI Insight. 8 (10), e164954. 10.1172/jci.insight.164954 PMC10322690 37014713

