# Supplementary material for: Generation of 3 patient induced Pluripotent stem cell lines containing SORD mutations linked to a recessive neuropathy
Source: Stem Cell Res. Author manuscript; Available in PMC 2024 Aug 1. (PMC11259078; doi:10.1016/j.scr.2024.103449)
Supplement: 1 [file NIHMS2002940-supplement-1.pdf]

Cell Line Characterization

Cell Line ID: sord1DM
Passage #: 14
Specimen Type: Human iPSC Culture
Indication for Study: Routine Culture QC

Test Code: 100
Account #: NA
PO #: 0000327662

Date Received: 8/14/2020
Date Reported: 8/24/2020
Time in Culture: 3 Days

Banding Technique: GTL **Band Resolution:** Good
Metaphases Counted: 20 Analyzed: 7 Karyotyped: 2

Lab #: CLG-39964
PI: Mario Saporta
Contact Person: Christopher Yanick
Email: cxy256@miami.edu
Address:
University of Miami
1420 NW 9th Avenue, Room 230A
Miami, Florida 33136

Additional copies sent to:

RESULTS: 46,XX[20] Apparently NORMAL Human Female Karyotype

Non-clonal Aberrations: None

INTERPRETATION:

Cytogenetic analysis was performed on twenty G-banded metaphase cells from human cell line sord1DM p14, and all twenty cells demonstrated an apparently normal female karyotype.

Case name: CLG-39964

Cell Line Name: sord1DM p14

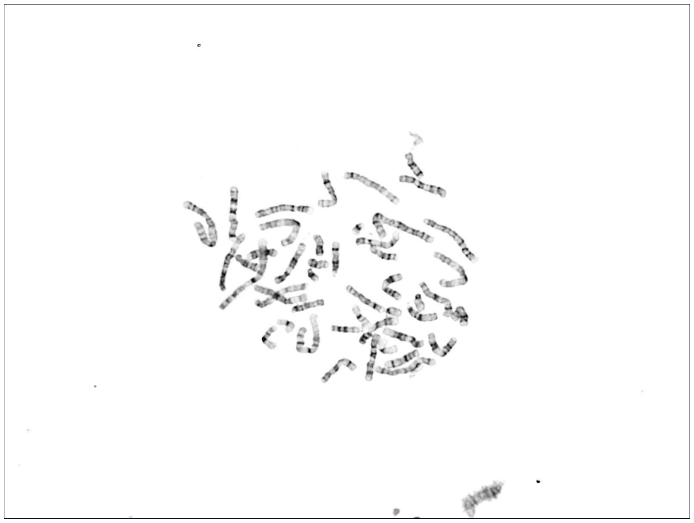

Result: 46,XX

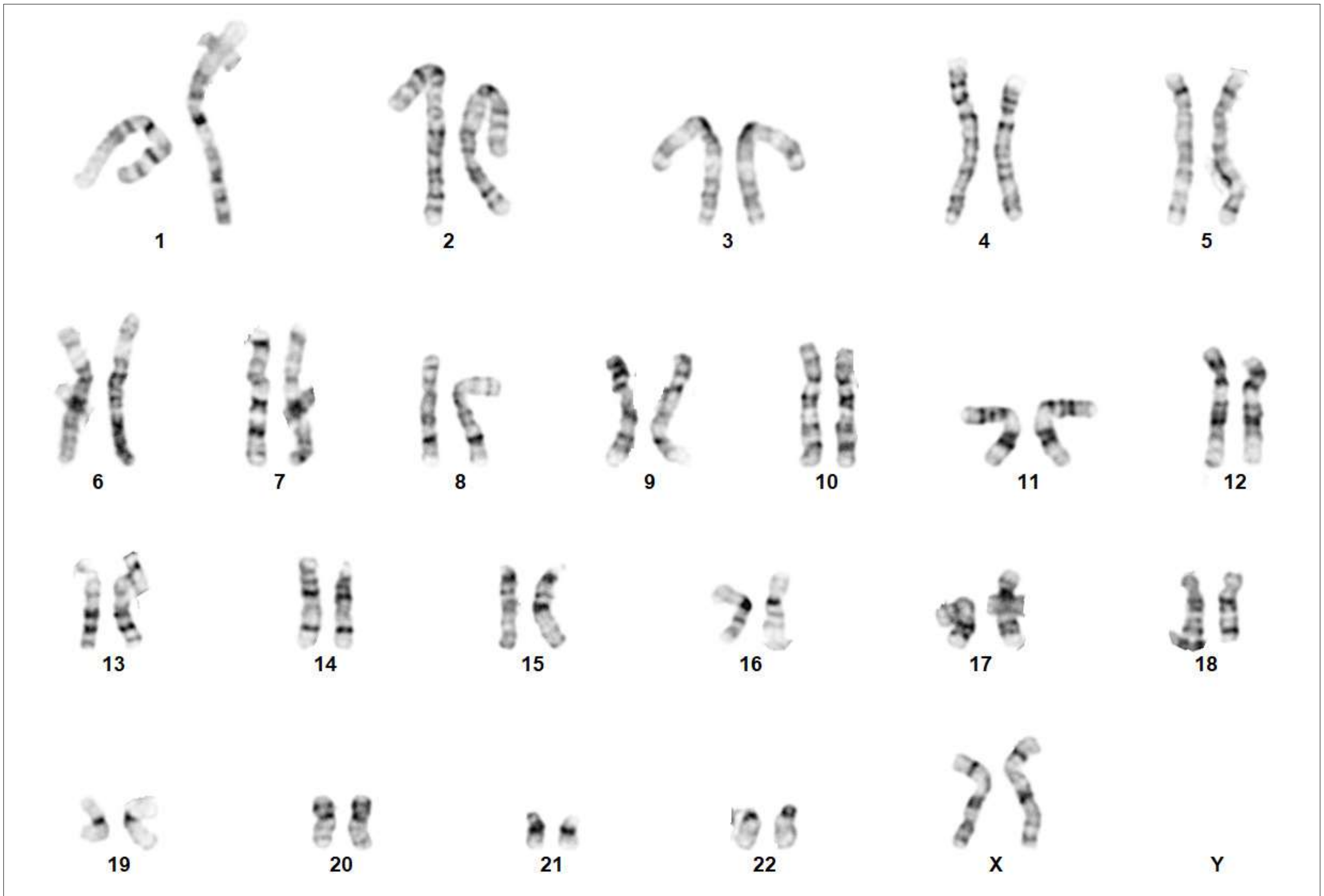

Case: CLG-39964 Slide: 39964-1_3 Cell: O14/4_cell 370

Cell Line GENETICS®

Cell Line Characterization

Cell Line ID: sord1GB
Passage #: 14
Specimen Type: Human iPSC Culture
Indication for Study: Routine Culture QC

Test Code: 100
Account #: NA
PO #: 0000327662

Date Received: 8/14/2020
Date Reported: 8/24/2020
Time in Culture: 3 Days

Banding Technique: GTL
Band Resolution: Good
Metaphases Counted: 20 Analyzed: 7 Karyotyped: 2

Lab #: CLG-39965
PI: Mario Saporta
Contact Person: Christopher Yanick
Email: cxy256@miami.edu
Address:
University of Miami
1420 NW 9th Avenue, Room 230A
Miami, Florida 33136

Additional copies sent to:

RESULTS: 46,XX[20] Apparently NORMAL Human Female Karyotype

Non-clonal Aberrations: None

INTERPRETATION:

Cytogenetic analysis was performed on twenty G-banded metaphase cells from human cell line sord1GB p14, and all twenty cells demonstrated an apparently normal female karyotype.

Case name: CLG-39965

Cell Line Name: sord1GB p14

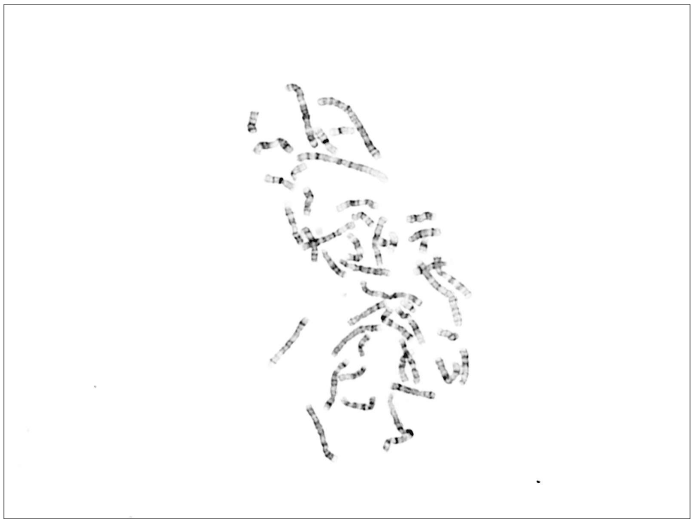

Result: 46,XX

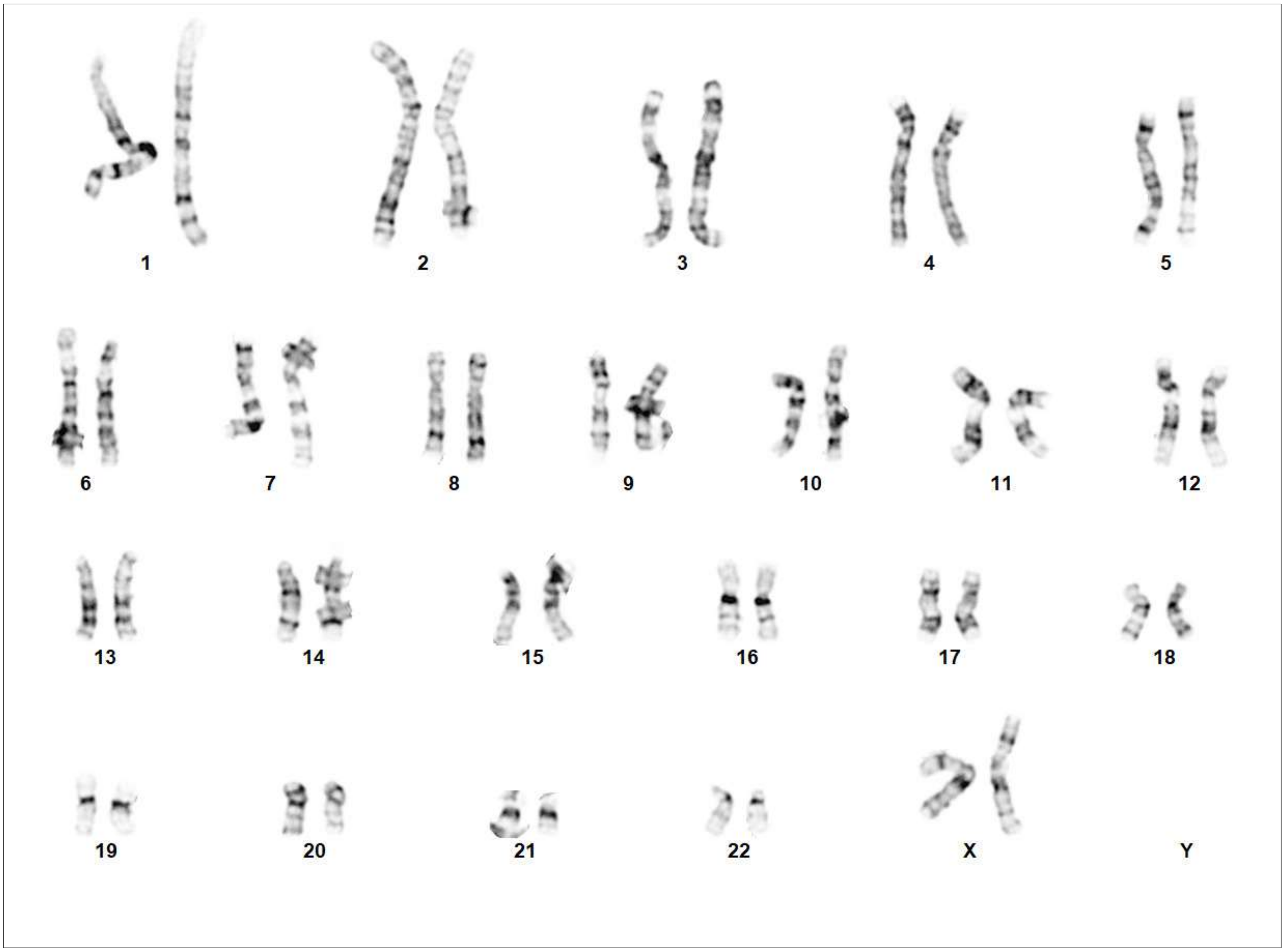

Case: CLG-39965 Slide: 39965-1_4 Cell: P18/3_cell 574

Cell Line Characterization

Cell Line ID: IIsord1RV
Passage #: 14
Specimen Type: Human iPSC Culture
Indication for Study: Routine Culture QC

Test Code: 100
Account #: NA
PO #: 0000327662

Date Received: 8/14/2020
Date Reported: 8/24/2020
Time in Culture: 3 Days

Banding Technique: GTL **Band Resolution:** Good
Metaphases Counted: 20 Analyzed: 7 Karyotyped: 2

Lab #: CLG-39966
PI: Mario Saporta
Contact Person: Christopher Yanick
Email: cxy256@miami.edu
Address:
University of Miami
1420 NW 9th Avenue, Room 230A
Miami, Florida 33136

Additional copies sent to:

RESULTS: 46,XY[19] Apparently NORMAL Human Male Karyotype

Non-clonal Aberrations: 45,XY,-18(one cell)

INTERPRETATION:

Cytogenetic analysis was performed on twenty G-banded metaphase cells from human cell line IIsord1RV p14. Nineteen cells demonstrated an apparently normal male karyotype, and one cell demonstrated a non-clonal chromosome aberration (listed above), which is most likely an artifact of culture.

RUO - Research Use Only

Case name: CLG-39966

Cell Line Name: IIsord1RV p14

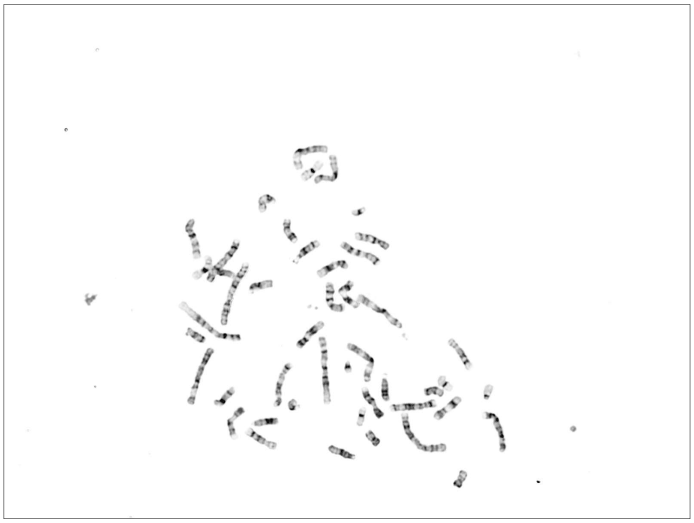

Result: 46,XY

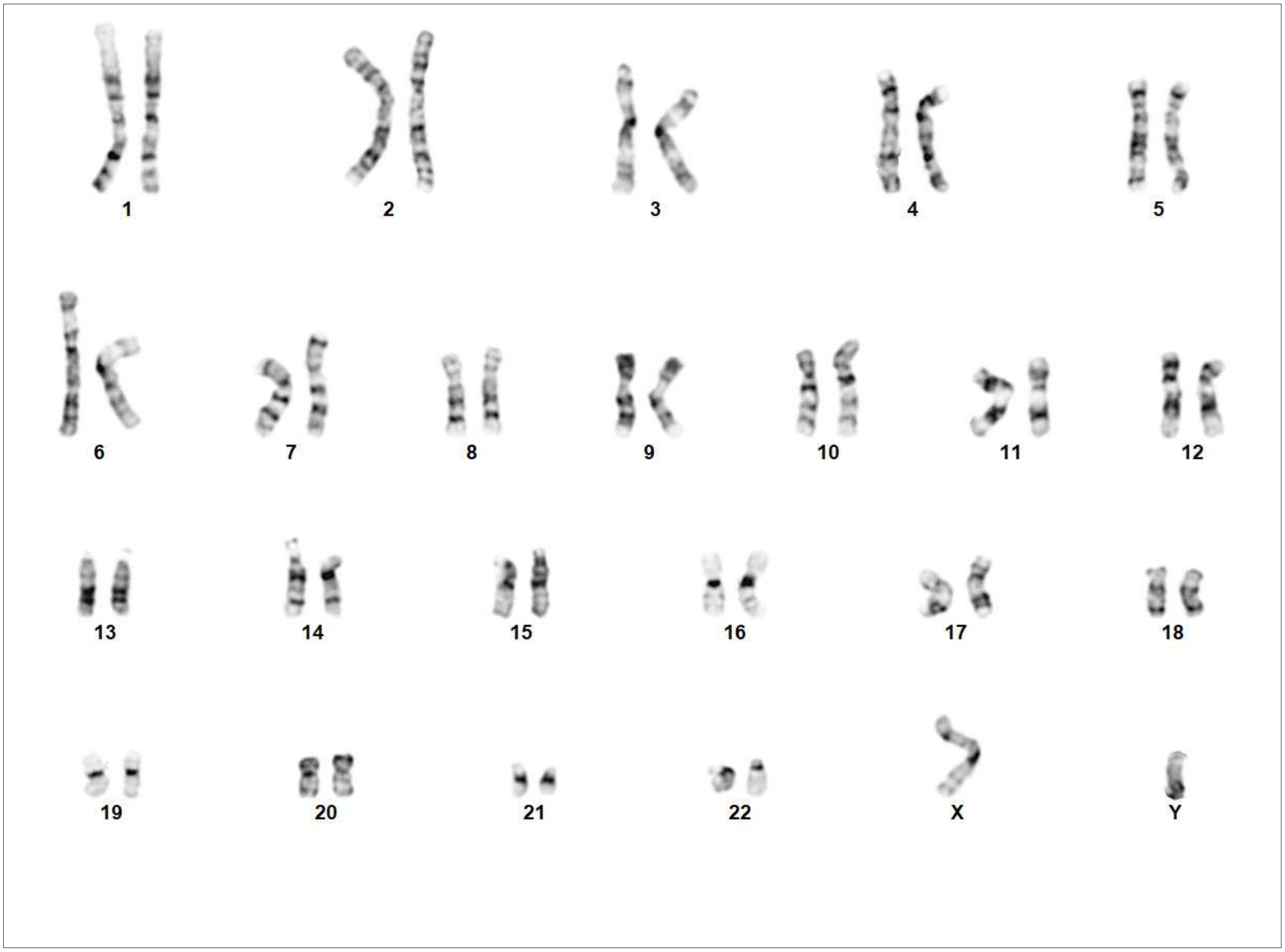

Case: CLG-39966 Slide: 39966-3_5 Cell: O20/0_cell 463
